# Stimulation of Immature Lung Macrophages with Intranasal Interferon Gamma in a Novel Neonatal Mouse Model of Respiratory Syncytial Virus Infection

**DOI:** 10.1371/journal.pone.0040499

**Published:** 2012-07-06

**Authors:** Kerry M. Empey, Jacob G. Orend, R. Stokes Peebles, Loreto Egaña, Karen A. Norris, Tim D. Oury, Jay K. Kolls

**Affiliations:** 1 Department of Pharmacy and Therapeutics, School of Pharmacy, University of Pittsburgh, Pittsburgh, Pennsylvania, United States of America; 2 Department of Pediatrics, Director, Richard King Mellon Foundation Institute for Pediatric Research, Children's Hospital of Pittsburgh, Pittsburgh, Pennsylvania, United States of America; 3 Department of Immunology, School of Medicine, University of Pittsburgh, Pittsburgh, Pennsylvania, United States of America; 4 Department of Pathology, School of Medicine, University of Pittsburgh, Pittsburgh, Pennsylvania, United States of America; 5 Department of Medicine, Division of Pulmonary, Allergy, and Critical Care medicine, Vanderbilt University School of Medicine, Nashville, Tennessee, United States of America; University of Liverpool, United Kingdom

## Abstract

Respiratory syncytial virus (RSV) is the leading cause of bronchiolitis and viral death in infants. Reduced CD8 T-cells and negligible interferon gamma (IFNγ) in the airway are associated with severe infant RSV disease, yet there is an abundance of alveolar macrophages (AM) and neutrophils. However, it is unclear, based on our current understanding of macrophage functional heterogeneity, if immature AM improve viral clearance or contribute to inflammation and airway obstruction in the IFNγ-deficient neonatal lung environment. The aim of the current study was to define the age-dependent AM phenotype during neonatal RSV infection and investigate their differentiation to classically activated macrophages (CAM) using i.n. IFNγ in the context of improving viral clearance. Neonatal and adult BALB/cJ mice were infected with 1×10^6^ plaque forming units (PFU)/gram (g) RSV line 19 and their AM responses compared. Adult mice showed a rapid and robust CAM response, indicated by increases in major histocompatibility complex class II (MHC II), CD86, CCR7, and a reduction in mannose receptor (MR). Neonatal mice showed a delayed and reduced CAM response, likely due to undetectable IFNγ production. Intranasal (i.n.) treatment with recombinant mouse IFNγ (rIFNγ) increased the expression of CAM markers on neonatal AM, reduced viral lung titers, and improved weight gain compared to untreated controls with no detectable increase in CD4 or CD8 T-cell infiltration. *In vitro* infection of J774A.1 macrophages with RSV induced an alternatively activated macrophage (AAM) phenotype however, when macrophages were first primed with IFNγ, a CAM phenotype was induced and RSV spread to adjacent Hep-2 cells was reduced. These studies demonstrate that the neonatal AM response to RSV infection is abundant and immature, but can be exogenously stimulated to express the antimicrobial phenotype, CAM, with i.n. rIFNγ.

## Introduction

Respiratory syncytial virus (RSV) is the primary cause of infant bronchiolitis and the most frequent cause of viral death in infants worldwide. According to the World Health Organization, there are 64 million cases of RSV each year resulting in 160,000 deaths globally. In the United States, annual RSV infection results in approximately 1.5 million outpatient visits among children <5 years of age with 75,000–125,000 estimated hospitalizations related to RSV among children aged <1 year, emphasizing the importance of age at initial infection [1], [2]. Despite the global burden of RSV disease, there remains no vaccine and no effective treatment. Disease pathology has been linked to host immune responses, which differs markedly in infants and adults [3], [4]. The critical role of CD8 T-cell and interferon gamma (IFNγ) production in adult RSV clearance has been well described [5]–[8]. Conversely, severity of infant RSV infection coincides with a deficient adaptive cytotoxic T-cell response and negligible IFNγ production [4], [9]–[11]. In the absence of a mature and efficient lymphocyte response, viral clearance in the infant airway is thought to rely more heavily on immature innate immune responses mediated largely by macrophages and neutrophils [4]. However, strikingly little is known regarding the phenotype and function of immature infant alveolar macrophages (AM) in the skewed T-helper 2 (Th2) cytokine (IFNγ-deficient) lung environment.

Far from our original understanding that all macrophages are pro-inflammatory, the concept of macrophage functional heterogeneity has gained considerable ground over the past decade [12]. Classical activation of macrophages differentiated by IFNγ and Toll-like receptor (TLR)-binding pathogens, including RSV, was historically believed to be the only pathway of macrophage activation. Classically activated macrophages (CAM) are characterized by production of nitric oxide (NO), secretion of interleukin-12 (IL-12), IL-1, IL-6, macrophage inflammatory protein-alpha (MIP-1α), and monocyte chemotactic protein-1 (MCP-1), and increased expression of major histocompatibility complex class II (MHC II), CD86, CCR7, cyclooxygenase-2 (COX2) and reduction in mannose receptor (MR) expression [13]–[15]. This pro-inflammatory response increases intracellular killing of phagocytosed organisms and promotes recruitment of additional antimicrobial cells, often at the expense of increased tissue damage. Alternatively activated macrophages (AAM), induced by IL-4 and IL-13, promote tissue repair through clearance of apoptotic cellular debris [13]. They also secrete the anti-inflammatory cytokine, IL-10 and produce arginase-1 that competes with inducible nitric oxide synthase (iNOS), rendering them useless in the killing of intracellular pathogens. Shirey and colleagues recently published a model for the role of AAM during RSV infection in adult rodents, which showed an immediate increase in CAM followed by a later rise in AAM through secretion of IL-4 and IL-13 from AM themselves [16]. This model provides a timely and critical explanation regarding how Th2-type cytokines may be released in the absence of T-cell infiltration in the infant airway during RSV infection. Yet, the extent to which CAM expression and anti-viral function occur in the RSV-infected infant lung in the absence of T-cell-derived IFNγ remains unknown. It also remains unclear if the promotion of AAM without the balance of CAM expression in the Th2 skewed, RSV-infected infant lung would remain protective or promote immunopathogenesis due to delayed viral clearance. To this end, we tested the hypothesis that RSV infection would result in increased CAM expression in adult mice due to the abundance of lymphocyte-derived IFNγ, but CAM expression would be reduced in neonatal mice with negligible IFNγ production. We further hypothesized that intranasal (i.n.) IFNγ would increase neonatal CAM expression during RSV infection leading to a reduction in viral load and improved weight gain.

We used an immunologically relevant neonatal mouse model of RSV infection to more closely recapitulate the human infant airway and an *in vitro* macrophage cell (J774A.1) line to further link the functional response of CAM following IFNγ priming. Similar to human infants, our infant mouse model demonstrated reduced lymphocyte infiltration, deficient endogenous IFNγ production, and a dominant macrophage response following RSV infection. Compared to RSV infected BALB/c adult mice, neonatal mice did not express CAM and had delayed viral clearance. Following treatment with i.n. IFNγ, RSV-infected, neonatal AM showed significantly increased expression of CAM markers, expedited RSV clearance and improved weight gain with no apparent increase in CD4 or CD8 T cell lung infiltration or activation. *In vitro*, RSV infection alone induced an AAM phenotype in J774A.1 macrophages and mediated the spread of virus to Hep-2 cells. However, when macrophages were first primed with IFNγ, cells expressed a CAM phenotype and the viral spread to adjacent Hep-2 cells was significantly reduced.

## Materials and Methods

### Ethics

This study was carried out in strict accordance with the recommendations in the Guide for the Care and Use of Laboratory Animals of the National Institutes of Health. Mice were housed at The University of Pittsburgh Division of Laboratory Animal Resources. These animal experiments were approved by The University of Pittsburgh Institutional Animal Care and Use Committee (IACUC), approved protocol number 1103468 and mice were handled according to IACUC guidelines. All efforts were made to minimize animal suffering.

### Mice and viral preparation

Pathogen-free breeder BALB/cJ mice were purchased at 5–7 wks of age from The Jackson Laboratory (Bar Harbor, ME) and were maintained in specific pathogen-free facilities. Female mice were cohoused for 2 wks to synchronize estrus for timed pregnancies. Additional pathogen-free, non-breeder female BALB/cJ mice were purchased at 8 weeks of age from The Jackson Laboratory for infection and control adult groups described below. Guidelines were followed for the care and use of animals as indicated by our animal review board. The line 19 subtype of RSV was provided by Dr. Martin Moore, Emory University, Atlanta, GA. RSV line 19 was passed through 4 rounds of plaque purification for generation of master stocks; working stocks were propagated in HEp-2 cells (American Type Culture Collection) and titered in HEp-2 cells using standard hematoxylin-eosin (H&E) plaque assays, as previously described [17]. Viral stocks were quick-frozen in alcohol/dry ice and stored at −80°C. Uninfected HEp-2 cell cultures were similarly processed to obtain a control, virus-free, mock preparation, referred to as cell lysate or mock. Viral stocks and HEp-2 cell lines were assessed to be free of mycoplasma and other common contaminants using the Plasmo Test Mycoplasma Detection Kit (InvivoGen) according to manufacturer's instructions. Lung titers were determined, as previously described, within one month of sterile removal and storage of infected lungs at −80°C [17]. Mice are considered neonates at less than 7 d of age and will be referred to in figures as pups.

### Histopathology

Heart-lung blocks were harvested 7 days post-infection (dpi) and fixed in 4% paraformaldehyde overnight. The lungs were transferred to 70% ethanol and then embedded in paraffin blocks. Tissue sections were stained with periodic acid-Schiff (PAS) to assess goblet cell hyperplasia as a measure of mucin expression. Slides were examined and scored by a single pathologist (T.O.) who was blinded to experimental groups. Individual airways were scored for goblet cell hyperplasia according to the following scale: 0 = no PAS positive cells; 1 = 1–25% PAS positive cells; 2 = 25–50% PAS positive cells; 3 = 50–75% PAS positive cells; 4 = 75–100% PAS positive cells. All airways involved in the tissue sections were scored.

### Infection protocol

Neonatal and adult mice were infected with a high dose/high volume (HD/HV) inoculation of RSV line 19. Between two to four days of age, neonates were infected intranasally under isoflurane anesthesia with a HD/HV of 1×10^6^ plaque forming unit (PFU)/gram (g) body weight of RSV line 19 in 10 microliters/g of body weight. Adult mice (8–9 weeks of age) were anesthetized with intramuscular (IM) ketamine 40 ug/g and xylazine 6 ug/g. They were simultaneously infected with 1×10^6^ PFU/g body weight of RSV line 19 in 9 microliters/g of body weight. Mock-infected mice were inoculated simultaneously with virus-free cell lysates in the respective volumes described above for neonates and adults. When held upright with the neck fully extended, the mice readily inhaled the stock virus placed over their nostrils with a micropipette. Following infection, mice were evaluated until full recovery from anesthesia. All adults and 92% (55 of 60) of neonates survived the infection protocol; this is similar to the survival rate for our standard volume (adults = 50 µl; neonates = 10–12 µl) infection protocols.

### IFNγ administration

Two groups of neonatal mice, two to four days of age, were infected with HD/HV RSV line 19. Recombinant murine IFNγ (rIFNγ) or diluent (0.1% mouse serum in PBS) was administered to neonatal mice intranasally as previously described [18]. Briefly, RSV-infected (RSV+) and mock-infected (mock) neonates received 16 ng/g rIFNγ (Peprotech, Rocky Hill, NJ) diluted in 10 µl of 0.1% mouse serum/PBS on 1, 3, 5, and 7 dpi. Groups are as follows: 1) RSV-infected/rIFNγ-treated = RSV/rIFNγ+; 2) RSV-infected/diluent only = RSV/rIFNγ−; 3) Mock-infected/rIFNγ-treated = mock/rIFNγ+; 4) Mock-infected/diluent only = mock/rIFNγ−. The mock/rIFNγ− control group was from a separate experiment with age-matched (two to four day-old) BALB/cJ mice.

### Isolation of lung interstitial and alveolar cells

Lung cells were prepared as previously described [19]. Briefly, adult and neonatal lungs were lavaged with five washes (for final volumes of 5 and 1.5 to 3 ml, respectively) of cold HBSS-EDTA (3 mM). Left lung lobes were used for viral titers; right lung lobes were used for flow cytometry analysis. Briefly, they were excised, weighed, minced, and enzyme treated at 37°C for 1 h in RPMI 1640 medium containing 3% fetal calf serum, 50 U/ml DNase (Sigma-Aldrich), and 1 mg/ml collagenase A (Sigma-Aldrich). Digested lung tissues were pushed through mesh screens to obtain a single-cell suspension. Red blood cells (RBCs) were lysed by treatment with a hypotonic buffer, and the cells were then resuspended in HBSS for enumeration and phenotypic analysis by flow cytometry.

### Flow cytometry

Surface protein expression and intracellular cytokine secretion were determined by flow cytometry. Isolated aliquots of 5×10^5^ to 10^6^ cells from lung lavage and digest were resuspended in staining buffer (1× PBS, 0.1% BSA, 0.02% sodium azide) and centrifuged at 1200 rpm for 8 min, at 4°C. After removal of supernatants, cells were blocked with anti-CD16/32 (BD Biosciences) for 15 minutes. Cells were stained with appropriate combination panels of fluorochrome-conjugated antibodies (Abs) specific for murine CD11b-perCpCy5.5, CD86-v450, major histocompatibility complex (MHC) class II (MHC II; I-a^d^)-FITC, and CD4-FITC, and CD62L-V450 (BD Biosciences, Mountain View, CA); CD11c-PE-Cy7, CD8a-APC, and CD44-PE (eBiosciences, San Diego, CA); and (mannose receptor) MR-Alexa Fluor 647 (AbD Serotec), as well as their isotype controls. Prior to staining MR, cells were treated with Leucoperm according to manufacturer's instructions (AbD Serotec). Cells were washed (2×) with staining buffer, fixed with 0.5% paraformaldehyde and analyzed within 12 hours; analysis showed all tandem dyes remained intact following fixation. Labeled cells were analyzed with an LSRII flow cytometer system (BD Biosciences). For figures in which cell numbers are given, percentage of the gated cell subset for each sample was multiplied by the number of cells manually counted by hemocytometer. FlowJo software (Tree Star Inc. Ashland, OR) was used to analyze the data.

### Cytokine and chemokine analysis

Neonatal and adult lungs were lavaged with 1.5–3 mls and 5 ml respectively, of HBSS/3 mM EDTA; bronchoalveolar lavage fluid (BALF) was stored at −80°C until analysis. Lung supernatants were assayed for IFNγ, IL-12p40, IL-6, TNFα, MCP-1, IL-10, and IL-4 using mouse inflammation multiplex assays (Bio-Rad, Hercules, CA) according to manufacturer's instructions. The plates were read using a Luminex® 200™ Total System machine (Luminex Corp, Austin, Tx). The data were analyzed using the LDS1.7 Software.

### Infection center assay

The mouse macrophage cell line, J774A.1 (TIB-67) was purchased from ATCC and cultured according to recommended specifications. Cells were seeded in 12-well plates at a concentration of 1×10^4^ cells/well and allowed to adhere. In separate experiments, cells were primed with 165 ng/ml of rIFNγ, 10 ng/ml each of IL-4/IL-13 (Peprotech, NJ), or media only for 24 hours after which time the priming solutions were removed. The cells were then immediately infected with RSV line 19 (generous gifts from Dr. Stokes Peebles, Vanderbilt University and Dr. Martin Moore, Emery University) at a multiplicity of infection (MOI) of 0.05. The cells were rocked for 3 hours, then overlaid with Hep-2 cells and incubated for 5 hours. The cells were then covered with methylcellulose and incubated for 5 days at which time a standard H&E staining protocol was followed. Control wells consisted of C1) BLANK, C2) J774A.1+, RSV+, rIFNγ−, C3) J774A.1−, RSV+, rIFNγ+, C4) J774A.1+, RSV−, rIFNγ+.

### Real-Time Polymerase Chain Reaction (PCR)

J774A.1 macrophages were seeded in 12-well plates at a concentration of 1×10^4^ cells/well and allowed to adhere as described for the infection center assay. In separate experiments, cells were infected with RSV line 19 alone at a MOI of 3 or were first primed with 100 ng/ml of rIFNγ, prior to RSV infection; control wells received media only in all experiments. mRNA was isolated from cells harvested at 24 hour and 72 hours post-infection using RNeasy Mini Qiagen Kit (Life Technologies, NY) and quantified using a NanoDrop® spectrophotometer (Invitrogen, NY). The mRNA was reverse transcribed to cDNA using a Superscript III First-strand synthesis Supermix for qRT-PCR kit (Life Technologies, NY) and quantified on a 7500 ABI Fast RT-PCR system (Life Technologies). Pre-mixed Taqman primer and probes using a Fam/Tamara reporter/quencher combination were purchased from ABI specific for Arginase-1, mannose receptor (MRC-1), FIZZ1, iNOS, and COX-2. Results are represented as a relative increase from media only or from media+IFNγ, using the delta, delta ct method indicating fold change over the house keeping gene (GAPDH). Data are compared to control wells treated with media only using a paired T-test for 3–4 wells per group.

### Statistical Analysis

Data are expressed as the mean ± SD of at least five mice per group and each experiment was repeated at least twice. Statistics were performed using GraphPad Prism 5 Software (La Jolla, CA). A two-way ANOVA was used to compare differences among data collected at days post-infection between neonatal and adult groups and between IFNγ-treated and IFNγ- neonatal groups and J774A.1 primed or unprimed groups, followed by a Bonferroni post-test. A paired student's t-test was used to compare viral titers for IFNγ-treated versus non-treated pups to show a less rigorous analysis, as the analysis at each time point was not dependent on the previous time point. A linear regression analysis was used to compare the slopes of RSV versus mock infected pup weights as a percent change of original weight over time using GraphPad Prism 5 Software. A Student t-test was used to determine the difference between average values of the slopes between the two groups; R^2^ values for each fitted line was ≥0.9.

## Results

### Infection of neonatal BALB/cJ mice with RSV line 19 effectively models human infant RSV infection

**Figure 1:**
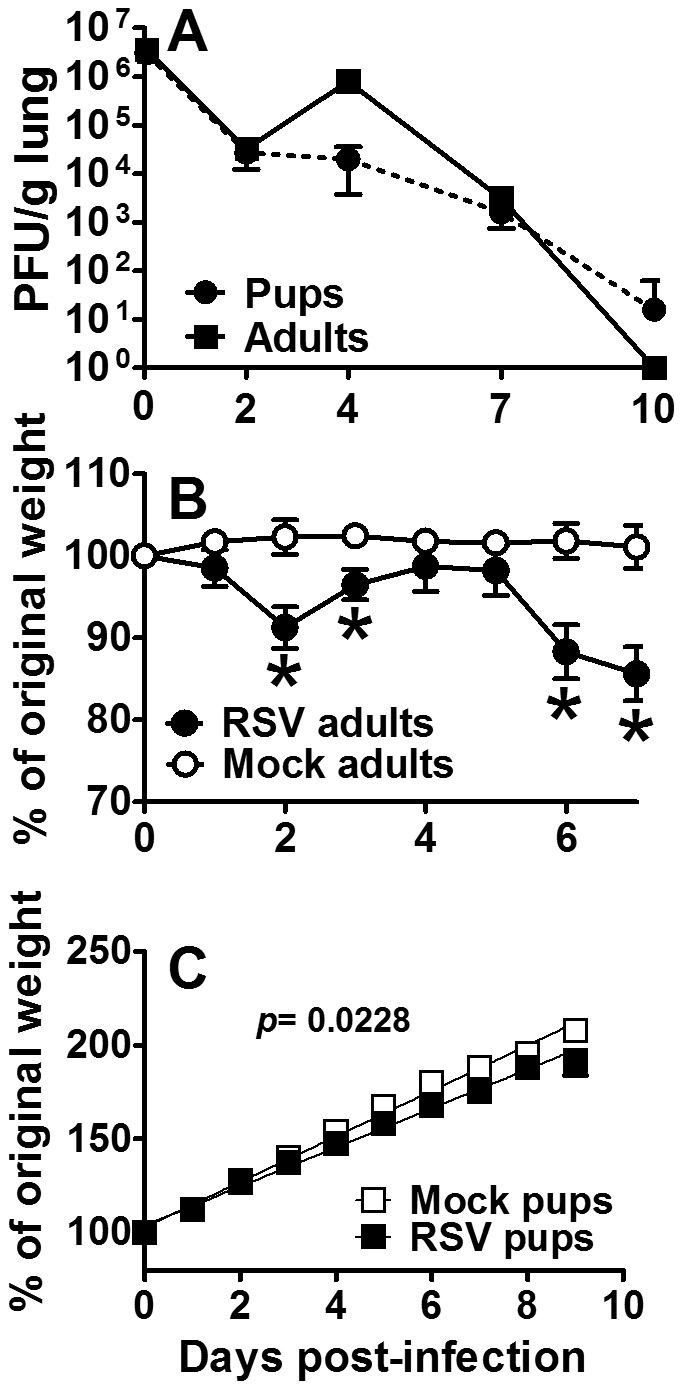
Effective infection of pup and adult mice with RSV line 19. Adult (8–9 wks old) and pup (2–4 days old) BALB/cJ mice received a HD/HV inoculum of RSV line 19 or cell lysate. Serial viral titers were determined by standard H&E plaque assay (A). Daily weights were measured in adults (B) and pups (C). For linear regression analysis, pup data are representative of 3 separate experiments with 15 pups per group. For viral titers and adult weights, data are representative of 3 experiments and points represent data for at least 5 mice per group ± SD. *, Significant compared with mock groups at p<0.05.

To validate our neonatal mouse model as an effective tool for evaluating human infant AM responses to disease, viral load was quantified from neonatal and adult BALB/cJ mouse lungs following RSV line 19 infection. Virus was delivered to the upper and lower airways (data not shown) using a high dose/high volume (HD/HV) inoculum of 1×10^6^ PFU/g of RSV in 9 µl and 10 µl/g of diluent in adults and neonates, respectively. Mice were inoculated at two to four days and eight to nine weeks of age in three separate experiments. Viral titers were quantified from left lung lobes on 0, 2, 4, 7, and 10 dpi from four to six mice per age group at each time point; the same dosing strategy was used in all subsequent experiments. Adult viral lung titers peaked at 4 dpi and become undetectable after 7 dpi (Fig. 1A). Neonatal lung titers showed persistent growth through 10 dpi, suggesting lower peak titers, but extended viral replication in infant compared to adult mouse airways. Daily weights were monitored for signs of illness. A characteristic bimodal weight loss pattern was observed in adult mice following RSV line 19 as is common with RSV A2 [20], yielding a peak weight loss of ∼20% total body weight by 7 dpi (Fig. 1B). To account for continuous weight gain in infant mice of about ∼1 g every 1–2 days, linear regression analysis was used. Weight data from each of three repeat experiments (15 pups per group) showed a moderate, but significant divergence in growth rates between RSV-infected and mock-infected neonatal mice (Fig. 1C).

**Figure 2:**
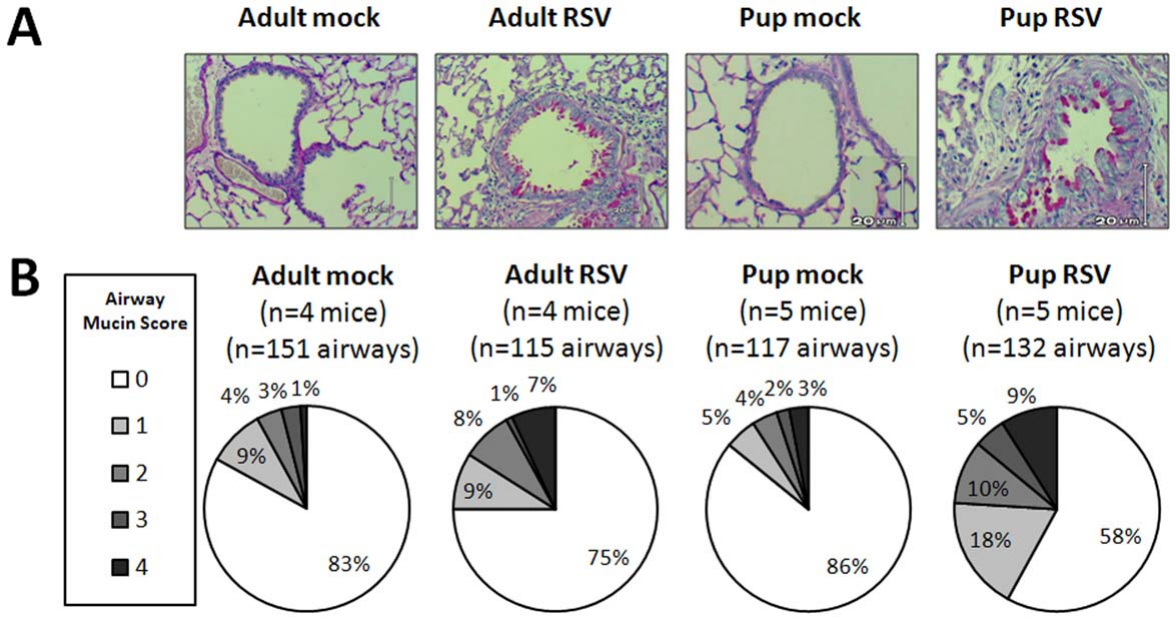
RSV line 19 induces pulmonary mucin expression in adult and pup BALB/cJ mice. Adult (8–9 wks old) and pup (2–4 days old) BALB/cJ mice received a HD/HV inoculum of RSV line 19 or cell lysate. The lungs were harvested at 7 dpi and sections were stained with PAS. Airways were scored 0 to 4 for PAS positivity (Materials and Methods). Twenty to 42 airways were scored per mouse. (A) Examples of airway mucin scores. Bright pink PAS-positive cells can be visualized in RSV-infected adult and pup sections. (B) Total numbers of mice and airway scored for each group are shown. The pie charts indicate the mucin scores ranked as a percentage of total airways from the respective groups.

Mucus production is a hallmark of severe infant RSV disease and has been shown to inhibit macrophage phagocytosis [21]. To determine if our neonatal mouse model of RSV infection demonstrated increased mucus production, we compared PAS-staining in RSV- and mock-infected neonatal and adult lungs. Lungs were harvested at 7 dpi, a time frame in which viral load is elevated and neonatal mouse weights were beginning to diverge among infected animals (Fig. 2A–B). A morphometric scale (Materials and Methods) shows more airways were producing mucin in RSV line 19-infected compared to mock-infected animals. RSV-infected neonatal mice had a greater relative expression of inspissated mucin lining the surface of bronchoepithelial cells. In contrast, adult mucin appeared intracellular, with little to no secretion observed. Together, these data demonstrate active RSV infection in infant BALB/c mice with increased mucus production and weight loss that serves as an effective model for testing immune modulating anti-RSV treatments, such as inhaled IFNγ.

### Innate immune cells, rather than CD8 T-cells, predominate during neonatal RSV infection

**Figure 3:**
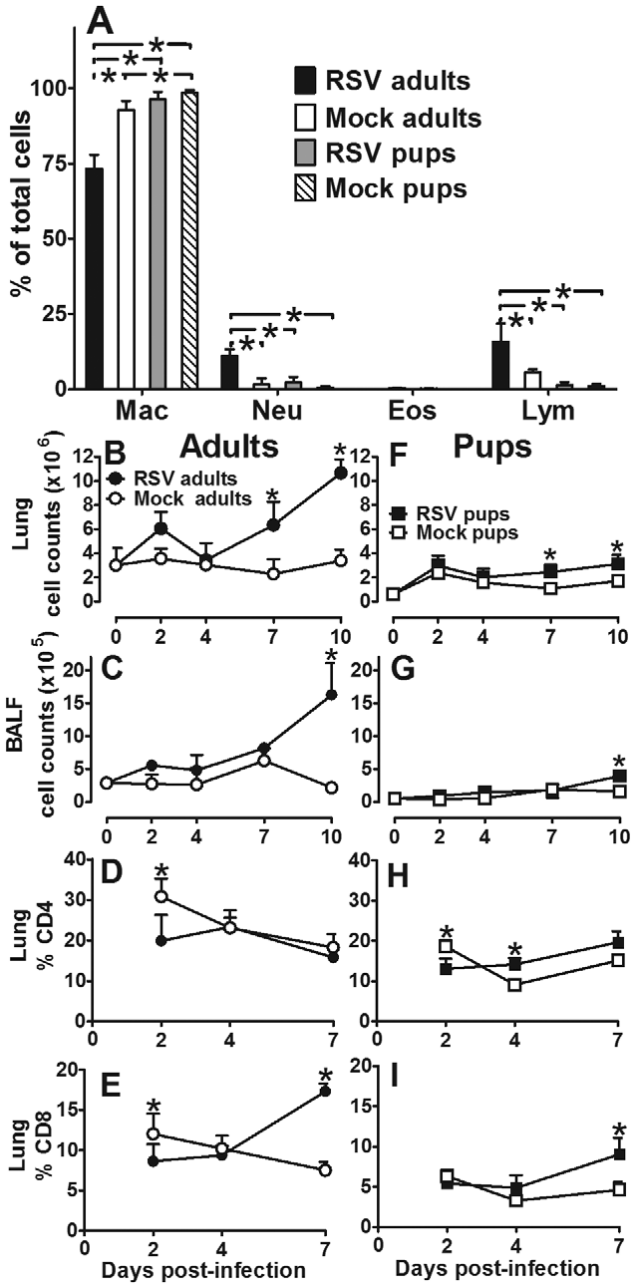
Age-dependent cellular response to RSV line 19. Adult (8–9 wks old) and pup (2–4 days old) BALB/cJ mice received a HD/HV inoculum of RSV line 19 or cell lysate. (A) At 7 dpi BALF was collected, H & E stained, and 200 cells per slide were counted using a 100× oil emersion objective lens. Data are depicted as percent of total cells that are macrophages (Mac), neutrophils (Neu), eosinophils (Eos), and lymphocytes (Lym). Cells were isolated from adult (B–C) and pup (D–E) BALF (C, G) and digested lung tissue (B, F). Total cells were quantified on 2, 4, 7, and 10 dpi by hemocytometer. Cells in adult (D–E) and pup (H–I) digested lung tissue were stained with anti-CD4 (D, H) and anti-CD8 (E, I) and analyzed by flow cytometry on 2, 4, and 7 dpi. Mean values ± SD are depicted, and statistical difference was defined as a *P* value.05 for differences between groups at the same time point (*); data are representative of two separate experiments.

Despite the well-established role of CD8 T-cells in RSV clearance reported in adult mice, recent evidence in human infants suggests innate immune cells, namely macrophages and neutrophils, may be more critical to viral clearance due to the absence of an efficient lymphocyte response [4]. To determine dominant cell population(s) during RSV infection, differential cell counts from BALF were determined at 7 dpi in four to six neonatal and adult animals. This time point represents the nadir of weight loss in infected adult animals, the divergence in weights among RSV- and mock-infected neonatal mice, and initial viral clearance among both age groups. Macrophages (96%) were the predominant cell type in RSV- and mock-infected neonatal mice (Fig. 3A). Conversely, RSV-infected adult animals responded with a significant increase in lymphocyte (16%) and neutrophil (11%) infiltration relative to mock-infected controls. To elucidate the effect of age on cellular infiltration following RSV infection, total cells were enumerated and compared in neonatal and adult BALF and lung tissue on 0, 2, 4, 7, and 10 dpi following RSV or mock infection. Total cells in neonate compared to adult lungs were significantly increased on 7 and 10 dpi in RSV- compared to mock-infected groups; however, the relative increase in the neonatal group was minimal (Fig. 3B, F). Cells did not enter the airways until 10 dpi in both neonatal and adult animals, and once again, cell numbers increased only minimally in infant airways (Fig. 3C, G), suggesting little cellular recruitment to the lung. To better understand the age-dependent infiltration of T-cells entering the lung at 7 dpi, neonatal and adult lungs were harvested following RSV- or mock- infection for analysis by flow cytometry. Adult animals responded to RSV infection with a robust increase in the percent of CD8 T-cells compared to mock-infected animals by 7 dpi in both the lung and BALF; no increases in CD4 T-cells were detected (Fig. 3D–E). Conversely, neonatal mice showed a modest increase in percent CD4 T-cells and CD8 T-cells relative to adult animals infected with RSV (Fig. 3H–I). Together, these data demonstrate that adult animals have a more robust lymphocyte response to RSV infection compared to neonatal animals. Regardless of age, lymphocytes appeared to be retained in the lung and failed to reach significant levels in the alveolar space until 10 dpi.

**Figure 4:**
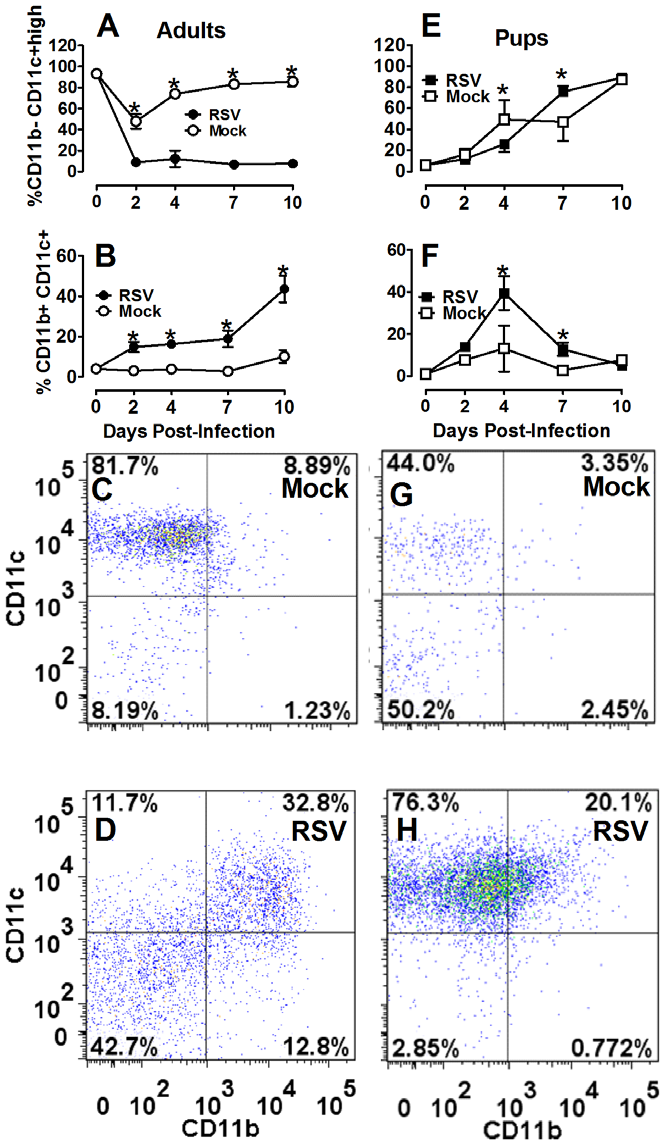
Recruitment and activation of neonatal antigen presenting cells is delayed following RSV infection. Adult (8–9 wks old) and pup (2–4 days old) BALB/cJ mice received a HD/HV inoculum of RSV line 19. The animals were lavaged at 0, 2, 4, 7, and 10 dpi. The percent of immune cell subtypes were analyzed by flow cytometry in adult (A–D) pup (E–F) BALF, including CD11b− CD11c+ high (nonlymphocyte gate) (A, E) and CD11b+ CD11c+ (nonlymphocyte gate) (B, F). Dot plots are representative of at least five mice per group for mock adults at 7 dpi (C), RSV adults (D), mock pups (G), and RSV pups (H). Mean values ± SD are depicted, and statistical difference was defined as a *P* value.05 for differences between groups at the same time point (*); data are representative of three separate experiments.

It has been postulated that AM are more important for viral clearance in the RSV-infected infant airway due to age-related deficiencies in CD8 T-cell responses, known to be critical for viral clearance in adult lungs [4], [5]. Thus, we interrogated the age-dependent cellular distribution of AM and lymphocytes following RSV infection. Neonatal and adult mice were infected with RSV or mock-infected as previously described. Single cell suspensions were stained with primary conjugated antibodies commonly used to discriminate AM (CD11b−/CD11c+high cells) and dendritic cells (DC) (CD11b+/CD11c+ cells) from lungs and BALF harvested at 0, 2, 4, 7, and 10 dpi and analyzed by flow cytometry [22]. Infection of adult BALB/cJ mice with RSV resulted in a rapid and continuous increase in the percent of DC with a simultaneous decrease in percent of AM which persisted through 10 dpi (Fig. 4A–D). Infection of neonatal mice with the same weight-based inoculum of RSV resulted in a transient peak in DC percentage at 4 dpi that rapidly subsided by 7 dpi with a concomitant increase in the percent of AM. By 10 dpi, AM approached 80% of total large cells in the airways of both RSV- and mock-infected neonatal animals suggesting the transient viral effects observed at 4 dpi were rapidly usurped by age-depended factors in order to achieve sufficient AM numbers (Fig. 4E–H). Together these data confirm that AM are the predominant cell type in the infant mouse model of RSV infection.

### Differential expression of cytokines following RSV infection indicates an early anti-inflammatory lung environment in infant compared to adult mice

**Figure 5:**
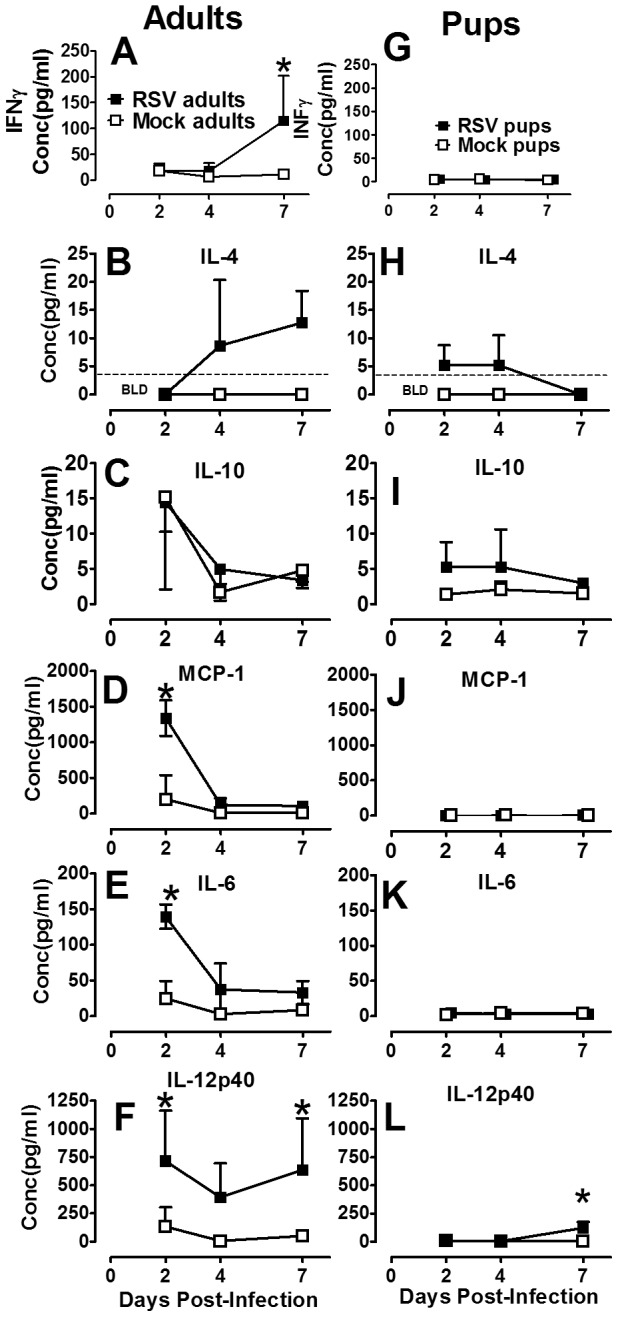
Neonatal mice produced early anti-inflammatory cytokines in response to RSV. Adult (8–9 wks old) and pup (2–4 days old) BALB/cJ mice were infected with HD/HV RSV line 19 or cell lysate, then lavaged with 5 ml or 1.5–3 ml of cold HBSS/EDTA, respectively at the indicated dpi. The first wash of each lavage was reserved and frozen for analysis by luminex multiplex assay. Adult (A–F) and pup (G–L) cytokine concentrations were measured at 2, 4, and 7 dpi. Dashed lines represents limits of quantification (LOQ) for the assay; if no dashed line given, all points are above the LOQ. Mean values ± SD are depicted, and statistical difference was defined as a *P* value .05 for differences between mock-infected animals at the same time point (*); data are representative of two separate experiments.

RSV disease and early age of infection are independently associated with a Th2-type cytokine response, hallmarked by increased IL-4 and/or reduced IFNγ production [23], [24]. To better understand the age-dependent cytokine environment that drives AM differentiation or result from AM activation, cytokines were measured by Luminex analysis in neonatal and adult BALF harvested at 2, 4, and 7 dpi. Following RSV infection, adult mice produced a significant increase in IFNγ and a modest increase in IL-4 which appeared to coincide with increases in lymphocyte infiltration into the lung at 7 dpi (Fig. 5A–B). Increases in cytokines associated with CAM, including MCP-1, IL-6, and IL-12, increased rapidly after RSV infection in adult animals (Fig. 5D–E); no increase in IL-10 was observed. Conversely, infection of neonatal mice with the same weight-based dose of RSV resulted in an absence of IFNγ, coupled with increases in IL-4 and IL-10 (Fig. 5G–I). Moreover, neonatal mice produced no detectable increases in MCP-1 or IL-6 and delayed and reduced levels IL-12p40 in response to RSV infection (Fig. 5J–L). These data suggest lymphocyte-derived IFNγ and moderate IL-4 levels are produced in response to RSV infection in adult animals as well as a robust early pro-inflammatory response. In neonatal mice, IFNγ production is absent but IL-4 and IL-10 increase by 2 dpi, long before lymphocyte infiltration into the lung, suggesting a non-lymphocyte source of IL-4.

### Neonatal BALB/cJ mice express an immature, age-dependent AM phenotype following RSV infection

**Figure 6:**
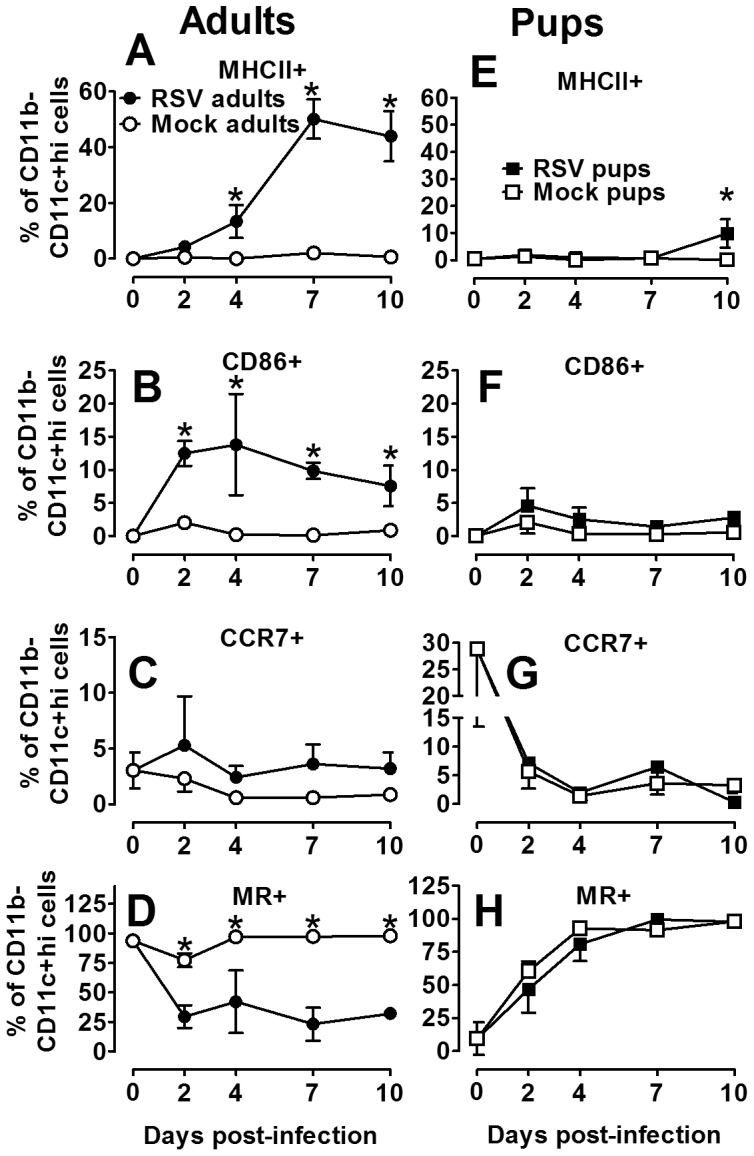
Neonatal BALB/cJ mice express an immature, age-dependent AM phenotype following RSV infection. Adult and pup BALB/cJ mice were infected with HD/HV RSV line19 or cell lysate. Cells were isolated from pup and adult BALF on 0, 2, 4, 7, and 10 dpi. The percent of immune cell subtypes were analyzed by flow cytometry in adults (A–D) pups (E–F), including MHC II (A, E), CD86 (B, F), CCR7 (C, G), and MR (D, H) on the CD11b− CD11c+ high gate. Mean values ± SD are depicted, and statistical difference was defined as a *P* value .05 for differences between mock-infected animals at the same time point (*); data are representative of two separate experiments.

The functional heterogeneity of AM is driven primarily by pathogens and environmental IFNγ and IL-4 ratios; disruption of influences that control the CAM and AAM balance could have severe consequences on airway inflammation and resolution of infection [25], [26]. Based on our findings that the ratio of IL-4 to IFNγ was greater in neonatal compared to adult animals following RSV infection, we sought to compare age-dependent AM phenotypes following RSV- or mock-infection. To determine the extent to which RSV induced CAM in neonatal versus adult BALB/cJ mice, the expression of surface markers indicative of CAM: MHCII, CD86, CCR7 and AAM: MR were quantified following isolation of AM from BALF harvested at 0, 2, 4, 7, and 10 dpi [12], [15], [27]. RSV infection of adult animals resulted in a robust increase in the expression of MHC II and CD86 and to a lesser degree CCR7; these marked changes in combination with a significant reduction in MR is suggestive of a CAM phenotype (Fig. 6A–D). Conversely, RSV infection of neonatal animals resulted in a striking absence of AM differentiation (Fig. 6E–H). A significant, but moderate increase in MHC II at 10 dpi suggested neonatal AM are activated in a delayed and deficient manner relative to adults during RSV infection (Fig. 6E). Moreover, an apparent ontologic shift toward a resting AAM phenotype was observed in both RSV- and mock-infected animals as indicated by reduced CCR7 and increased MR (Fig. 6G–H). When similar measurements were made on digested lung tissues, equivalent trends in MHC class II, CD86, CCR7, and MR expression were observed in tissue macrophages of adult and neonatal BALB/cJ mice infected with RSV or mock-infected (Fig. S1). Together, these data suggest RSV line 19 induces CAM in adult mice, but fails to effectively induce classical activation in immature neonatal AM, likely due to the absence of IFNγ.

### Inhaled rIFNγ expedites viral clearance in RSV-infected neonatal mice

**Figure 7:**
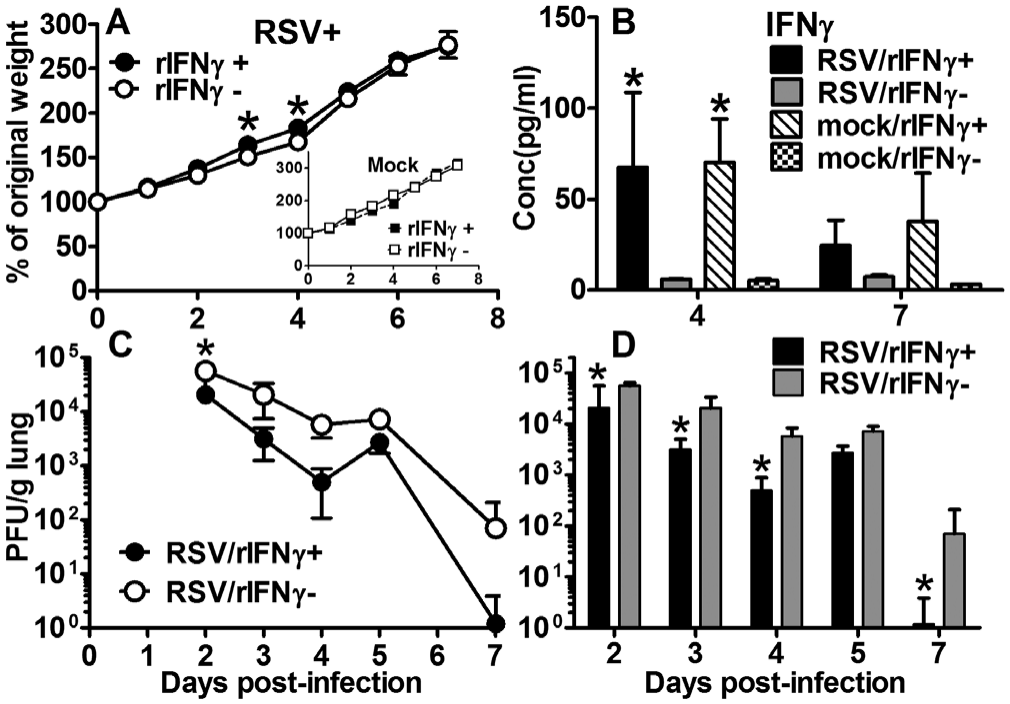
Inhaled rIFNγ reduces viral load in RSV-infected neonatal BALB/cJ mice. On 1, 3, 5, and 7 dpi pups received 16 ng/g of i.n. rIFNγ or diluent only. (A) Daily weight change compared to weight prior to infection was plotted; (B) IFNγ was measured from BALF by Luminex assay. Viral titers were measured from left lung lobes by H&E plaque assay on 2, 3, 4, 5, and 7 dpi. RSV titers were analyzed by a 2-way ANOVA and graphically represented with a line graph (C) and by paired t-test, which is graphically represented by a bar graph (D). Mean values ± SD are depicted, and statistical difference was defined as a *P* value .05 for differences between mock-infected animals at the same time point (*); data are representative of two separate experiments.

We previously observed delayed viral clearance in our neonatal BALB/cJ mouse model of RSV infection in the absence of endogenous of INFγ compared to adult animals. Thus, we sought to determine if treatment with i.n. rIFNγ would then expedite the RSV clearance in our infant RSV mouse model. Mice were RSV- or mock-infected between two to four days of age as previously described. Mice within each of these groups were treated with i.n. rIFNγ or diluent on 1, 3, 5, and 7 dpi. To assess potential toxicity due to rIFNγ, daily weights were compared among treated and untreated mice. Lungs and BALF were harvested at the indicated days pi. No weight loss in the RSV line 19 or mock-infected groups due to rIFNγ was observed; rather a significant increase in weight gain was observed in the RSV/rIFNγ+ group (Fig. 7A). Increased levels of IFNγ in BALF among treated mice indicated inhaled cytokine delivery was an effective method of administration (Fig. 7B). To determine the effect of rINFγ on RSV clearance, viral lung titers were quantified by plaque assay. A more conservative 2-way ANOVA was used to analyze the results indicating a significant reduction in viral burden at 2 dpi compared to untreated mice (Fig. 7C). Considering the apparent decrease in viral load observed at 4 and 7 dpi, separate paired t-tests were used to analyze viral titers at each time point, showing significant reductions in viral titers at 2, 3, 4, and 7 dpi (Fig. 7D). These data determined that inhaled delivery of rIFNγ reduced RSV lung titers by a log-fold reduction and effectively expedited viral clearance to within 7 dpi (Fig. 7C) compared to untreated animals, in which titers extended beyond 10 dpi (Fig. 1A).

### Inhaled rIFNγ increases CAM activation in RSV-infected neonatal BALB/cJ mice without T-cell activation

**Figure 8:**
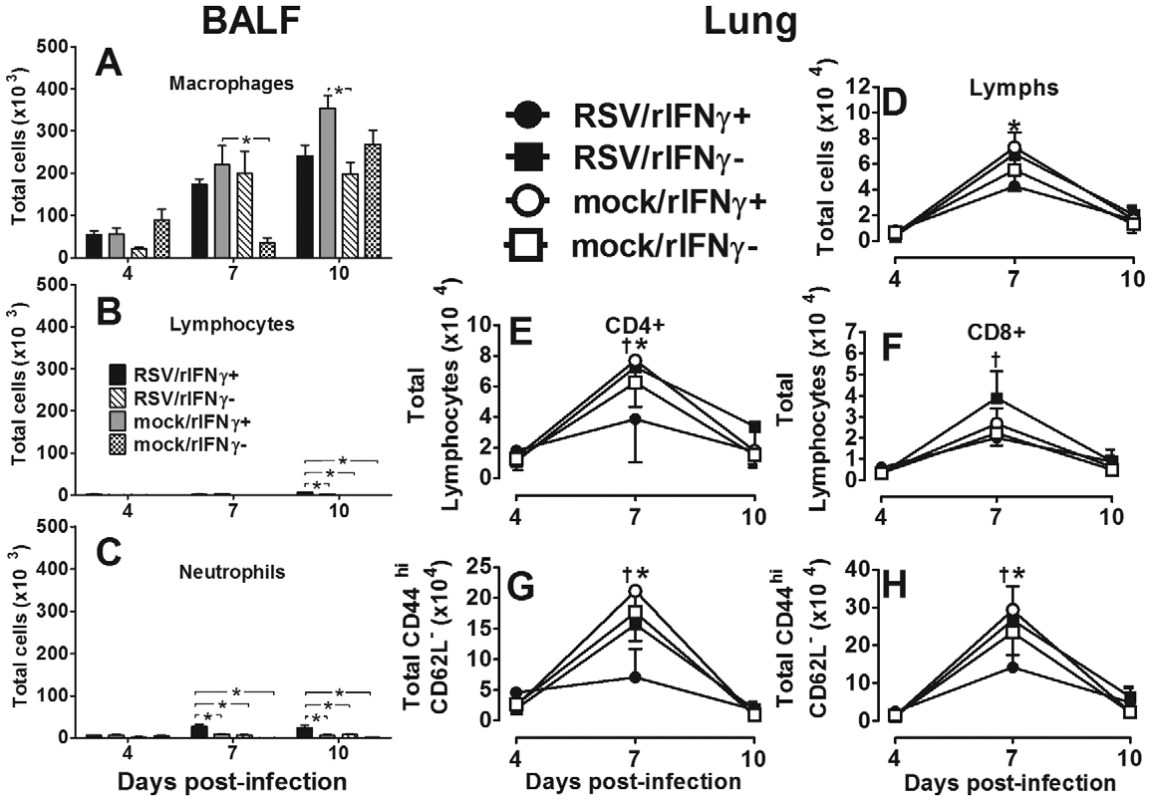
rIFNγ does not increase CD4 or CD8 T-cell activity in RSV-infected infant mice. Pup (2–4 days old) BALB/cJ mice received a HD/HV inoculum of RSV line 19 followed by 16 ng/g of i.n. rIFNγ (RSV/rIFNγ+) or diluent only (RSV/rIFNγ-) on 1, 3, 5, and 7 dpi. Control groups were mock-infected with cell lysate followed by16 ng/g of i.n. rIFNγ (mock/rIFNγ+) or diluent only (mock/rIFNγ −) on 1, 3, 5, and 7 dpi. BALF and lungs were collected at each time point. Cells were isolated from BALF, H & E stained, and 200 cells per slide were counted using a 100× oil emersion objective lens. Data are depicted as Total number of cells that are macrophages (Mac) (A), lymphocytes (Lym) (B), and neutrophils (Neu) (C); eosinophils were negligible. Lungs were enzyme digested, stained with fluorochrome-labled antibodies specific CD4, CD8, CD44, and CD62L, and analyzed by flow cytometry (E–H); all cells were first gated by small forward and side scatter (D). Bars represent data for at least five mice per group from two experiments. Mean values ± SD are depicted, and statistical difference was defined as a *P* value .05 for differences between groups at the same time point (*).

**Figure 9:**
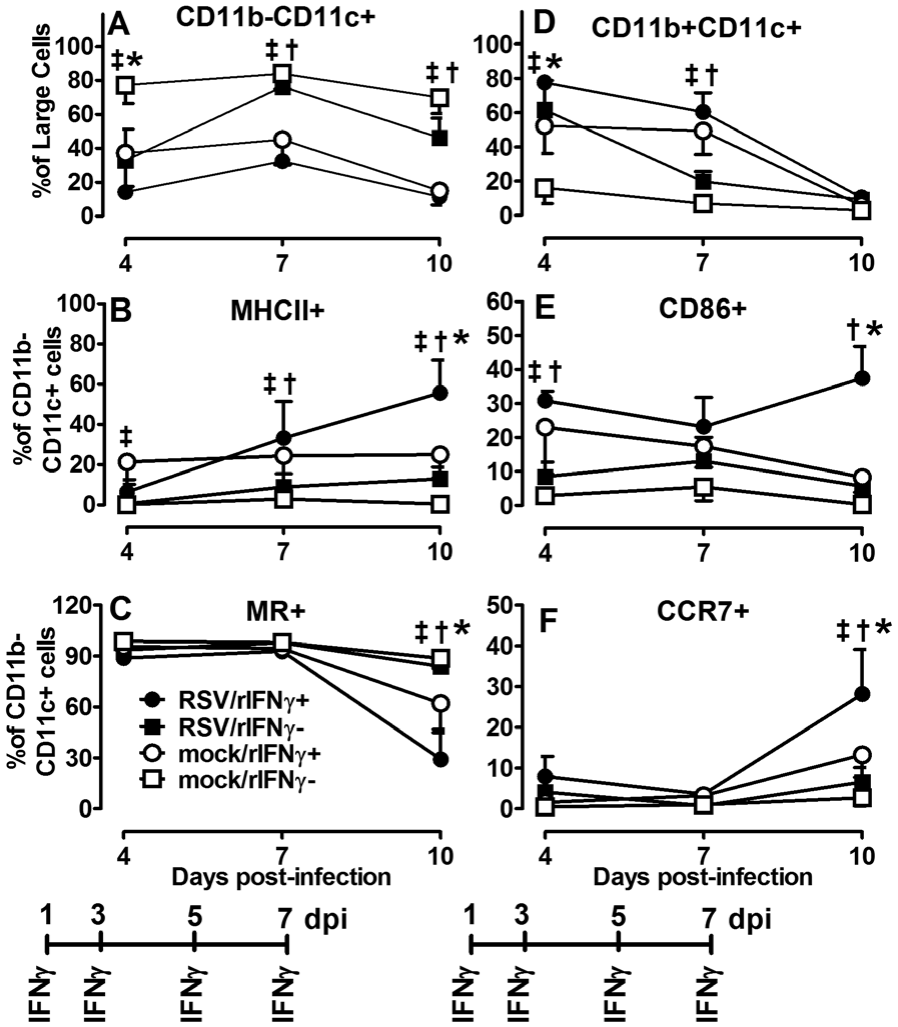
Inhaled rIFNγ increases CAM activation in RSV-infected neonatal BALB/cJ mice. Pup (2–4 days old) BALB/cJ mice received a HD/HV inoculum of RSV line 19 followed by 16 ng/g of i.n. rIFNγ (RSV/rIFNγ+) or diluent only (RSV/rIFNγ−) on 1, 3, 5, and 7 dpi. Control groups were mock-infected with cell lysate followed by 16 ng/g of i.n. rIFNγ (mock/rIFNγ+) or diluent only (mock/rIFNγ −) on 1, 3, 5, and 7 dpi. Cells were isolated from BALF on 4, 7, and 10 dpi and percent CD11b− CD11c+ (nonlymphocyte gate) (A), CD11b+CD11c+ (nonlymphocyte gate) (D), and MHC II, MR, CD86, and CCR7 (gated on CD11b− CD11c+ cells) (B, C, E, F) were determined by flow cytometry. Points represent data for ≥5 mice per group ± SD. (†) indicates *P*<0.05 for comparisons between RSV/rIFNγ+ and RSV/rIFNγ−; (‡) indicates p<0.05 for comparisons between mock/rIFNγ+ and mock/rIFNγ−; (*) indicates p<0.05 for comparisons between RSV/rIFNγ+ and mock/rIFNγ+.

RSV-infected infants are prone to Th2-type cytokine responses. Reduced IFNγ and increased IL-4 and IL-13 have been attributed to immature host immune responses combined with the RSV organism itself, thought to induce Th2 cytokines from CD4 T cells, mast cells, basophils, and monocytes [4], [28], [29]. Thus, despite its macrophage stimulating properties in mature adults, the extent to which i.n. IFNγ induces CAM in RSV-infected, neonatal lungs cannot be assumed and remains largely unknown. To assure i.n. rIFNγ wasn't depleting the intended target cell population, AM were analyzed in BALF harvested on 4, 7, and 10 dpi from two to four day-old mice infected with RSV or mock-infected and treated with rIFNγ or diluent on 1, 3, 5, and 7 dpi as described above. Cell differential counts showed the RSV/rIFNγ+ group maintained a higher total number of macrophages compared to the RSV/rIFN- group at 4 dpi. Compared to lymphocytes, macrophages remained a dominant cell population in the RSV-infected infant airway (Fig. 8A–C). Despite increased viral clearance in the rIFNγ-treated group, total T-cell infiltration and activation was not increased compared to untreated animals on or before the time of increased viral clearance (Fig. 8B, D–H). To determine extent of CAM in RSV- and mock-infected neonatal mice treated with i.n. rIFNγ in AM (CD11b−/CD11c+ cells), cells were isolated and processed for flow cytometry from BALF harvested at 4, 7, and 10 dpi. As previously described, MHC II, CD86, CCR7, and MR expression were quantified over time in treated and control animals. RSV-infected mice treated with rIFNγ had significantly increased expression of CD86 by 4 dpi, MHC II was increased by 7 dpi and by 10 dpi CD86, MHC II and CCR7 expression was significantly increased and MR was reduced compared to untreated mice, indicating induction of a CAM phenotype (Fig. 9A–C, E–F). A multiplex cytokine analysis was performed on cytokines commonly produced by CAM in the BALF of RSV/rINFγ+ animals compared to RSV/rINFγ- animals. Similar to adult animals infected with RSV in Figure 5D–F, RSV-infected neonatal mice treated with IFNγ had increased production of IL-12p40, IL-6, and TNFα (Fig. S2). Moreover, no increase in IL-4 or IL-10 was observed following IFNγ as was previously shown in untreated infant mice. These data demonstrate that exogenous treatment with inhaled rIFNγ can overcome immaturity of AMs in a neonatal mouse model of RSV infection as measured by increased CAM expression of CD11b−/CD11c+ cells, increased production of IL-12p40, IL-6, and TNFα, and expedited viral clearance.

### Priming RSV-infected J774A.1 macrophages with IFNγ changes the expression phenotype from AAM to CAM and reduces the spread of virus to adjacent Hep-2 cells

**Figure 10:**
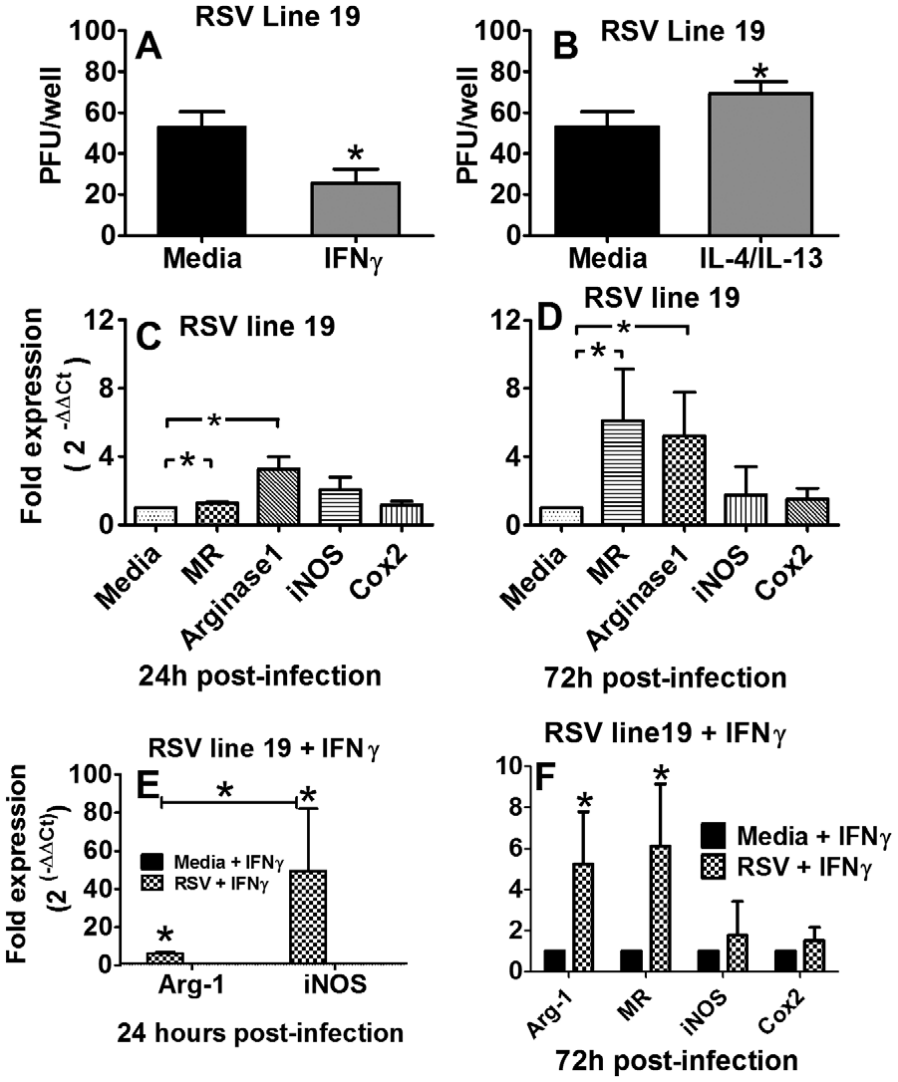
IFNγ-primed J774A.1 macrophages reduce the spread of RSV to adjacent cells. The mouse macrophage cell line, J774A.1, was cultured *in vitro* and primed with rIFNγ or media (A) or IL-4/IL-13 or media (B) prior to infecting with line 19 RSV for 3 hours. Infected cells were overlaid with Hep-2 cells and culture for 5 hours, covered with methylcellulose, incubated for 5 days and a standard H&E stain; plaques are reported as PFU per well. (C) Relative quantification by real-time PCR was performed on RNA extracted from cells infected with RSV line and presented as the fold expression over cells not infected with RSV; all assays were normalized to GAPDH. (D) Relative quantification by real-time PCR was performed on RNA extracted from cells primed with IFNγ and infected with RSV and presented as the fold expression over cells cultured in media alone (E–F); all assays were normalized to GAPDH. For infection center assays, data represent the mean ± SD of 12 wells per treatment and each control arm (Materials and Methods). For RT-PCR, data represent the mean ± SD of 4 wells per treatment and each control arm Statistical difference was defined as a *P* value .05 for differences between groups (*); data are representative of two separate experiments.

To determine if CAM contribute to RSV clearance, viral spread from infected J774A.1 murine macrophages to adjacent Hep-2 cells was examined. Briefly, J774A.1 macrophages were primed with rIFNγ, IL-4/IL-13 or media only, washed, infected with RSV line 19 at an MOI of 0.05 for 3 hours, and then overlaid with Hep-2 cells. After 5 hours, the cells were covered with methylcellulose and incubated for 5 days followed by standard H&E staining. Plaques represented RSV that had replicated in macrophages and infected adjacent Hep-2 cells. *In vitro* priming of J774A.1 macrophages with rIFNγ significantly reduced the number of RSV line 19 plaques compared to media-primed macrophages, in Hep-2 cells by at least 50 PFU per well (Fig. 10A). Conversely, in a separate experiment, J774A.1 macrophages primed with IL-4/IL-13 significantly increased the spread of virus to Hep-2 cells, presumably through a reduction in nitric oxide involved in intracellular killing of organisms (Fig. 10B). To assess changes in iNOS expression and other markers often used to discriminate between CAM and AAM, real-time PCR was used to measure message expression in J774A.1 cells after RSV infection (Fig. 10C). Greater increases in the expression of MR and arginase-1 compared to Cox-2 and iNOS indicate AAM differentiation in J774A.1 cells infected with RSV after 24 hours. By 72 h pi a greater increase in fold expression of MR and arginase-1 over iNOS and Cox2 was observed (Fig. 10D). When J774A.1 macrophages were primed with IFNγ prior to RSV infection, iNOS expression increased markedly over arginase-1 after 24 h, indicating CAM differentiation (Fig. 10E), which likely contributed to the reduced spread of virus to adjacent Hep-2 cells. Interestingly, priming with IFNγ alone was not sufficient to differentiate CAM, suggesting that RSV binds TLR on macrophages and provides an adequate secondary signal for CAM differentiation in mature J774A.1 macrophages. By 72 h after IFNγ priming, Arginase-1 and MR once again increased, indicating AAM expression resumes (Fig. 10F). Together, these data show that treatment of neonatal mice with i.n. rIFNγ not only reduces RSV lung titers by ∼ a log-fold reduction/gram of lung, but also expedites RSV clearance by 3 days in infant mice without causing weight loss. *In vitro* data support our hypothesis that i.n. rIFNγ induces CAM in the presence of RSV infection and contributes to viral clearance.

## Discussion

The aim of the current study was to define the age-dependent AM phenotype during neonatal RSV infection and investigate their differentiation to CAM using i.n. rIFNγ in the context of improving viral clearance. To accomplish this, a neonatal mouse model of RSV infection was developed based on five critical criteria regarding severe human infant RSV disease: 1) severe disease occurs when virus enters the lower airway, 2) mucus production is increased, 3) lymphocyte infiltration is minimal, 4) IFNγ production is negligible, and 5) there is an abundance of AM [4], [30]–[32]. The HD/HV inoculum is not a physiologic method of human infant infection; however, in our neonatal mouse model, this method introduced more virus to the lower airway compared to standard low volume methods which leaves more virus in the upper airway and nasal septum (data not shown). A similar method using ^99m^ Technetium sulfur colloid in four-week-old mice also demonstrated improved delivery to the lungs compared to the nasopharynx [17]. Moreover, in adult animal models, Lukacs et al, showed the RSV line 19 subtype induced significantly more mucus production than the A2 strain commonly studied in rodent models [30], [32]. Using these strategies, our neonatal mice maintained consistent viral lung titers compared to adult mice, which coincided with reduced weight gain (Fig. 1A–C) and increased PAS staining in neonatal and adult lungs (Fig. 2). These findings confirm that this model is an effective tool for studying immature infant host responses to RSV infection and targeted immune modulating RSV treatment strategies.

CD8 T-cells are critical in the response to RSV infection leading to both viral clearance and illness in adult mouse models [5]. In our neonatal mouse model we showed that macrophages were dominant in the infant airway with markedly reduced levels of CD8 T-cells compared to adults. Interestingly, adult animals had a predominant CD8 T-cell response, whereas infant mice did not. Coupled with significantly increased levels of IFNγ and IL-12p40, these data indicate a strong anti-viral cytotoxic T-cell response in adult animals, which was markedly reduced in neonatal mice. This immature lymphocyte response has been implicated in the dominant AM and neutrophil response observed in human infants with severe RSV disease [4], [9]. Welliver and colleagues analyzed lung tissue from infants with fatal cases of RSV and showed almost a complete absence of CD8 T-cells and NK cells coupled with an abundance of macrophages and neutrophils [4]. A similar study by Johnson and colleagues evaluated the histopathology of four fatal, untreated human infants with acute RSV infection [31]. They observed a majority of AM around the bronchioles, alveolar interstitium and airspaces. In our study, direct comparison of RSV-infected neonatal and adult animals emphasized the deficient lymphocyte and abundant AM response among neonatal mice and corroborates the findings in human RSV-infected infants (Fig. 3–4) [4]. Based on differential cell counts in the BALF at 7 dpi, 16% of the total cells in the adult BALF were lymphocytes compared to 1% in neonatal BALF. Similarly, there were significantly more CD8 T-cells in RSV- infected (17%) compared to mock-infected (7%) adult lungs versus neonatal RSV-infected (9%) and mock-infected (4%) lungs by 7 dpi, which coincided with an overall reduction in total cellular airway infiltration (Fig. 3). While some lymphocyte infiltration into infant lungs was apparent following RSV infection (albeit less than adults) the cells did not appear to migrate into the airway. Delayed migration of T-cells into infant airways was also recently reported by Lines and colleagues in a neonatal mouse model of influenza virus, indicating an age-dependent delay in cellular migration to the alveolar space during infection; despite i.n. administration of exogenous IFNγ, neonatal T cells could not be drawn into the airway before two weeks post-infection in this model [33].

Lymphocyte-derived cytokines, IFNγ and IL-4, increased in adult BALF in concert with the infiltration of lymphocytes into the lung at 7 dpi; however, IFNγ levels did not increase in RSV-infected compared to mock-infected neonatal mice. Interestingly, neonatal IL-4 levels increased very early by 2 dpi indicating that although production was not significantly increased, it had to be from a non-lymphocyte source. Shirey et al. recently published evidence in adult rodents suggesting that AM themselves are capable of IL-4 and IL-13 production, leading to the continuous promotion of an AAM phenotype and increased IL-10 production. While we did not observe an increase in IL-13 production, we did observe an early increase in the anti-inflammatory cytokine IL-10 in neonatal mice by 2 dpi, which was absent in adult animals. It is intriguing to consider that the innate host immune response is capable of initiating and maintain a Th2-type cytokine in the absence of T-cells. Increasing evidence to this effect is being realized, although the extent and the consequences in the infant airway remain unclear [34]. A balance of CAM and AAM in the absence of adaptive immunity may be critical to keep viral replication in check. Additional cytokines representative of CAM were increased early post-infection in adult animals compared with mock-infected animals, including MCP-1, IL-6, and IL-12p40. Neonatal mice failed to increase production of these cytokines, but for IL-12p40 which was delayed and reduced in its production relative to adult animals (Fig. 5). These cytokines were evaluated in total BALF and do not discriminate between macrophage-derived, or other acute response cell types, including DC, or airway epithelial cells. However, production of neonatal IL-12p40 does not occur until 7 dpi as DC are rapidly declining and AM are significantly greater than mock-infected controls. These data do not exclude the potential contribution of DC in the neonatal host immune response to RSV disease. However, together these data conclude that the neonatal AM phenotype and cytokine response are anti-inflammatory following RSV infection and their potential for targeted immunotherapy requires further investigation.

There is growing interest in AM as a potential vaccine or therapeutic target for severe neonatal RSV disease [35], [36]. However, understanding the age-dependent changes in AM functional heterogeneity, driven largely by IFNγ and IL-4, is critical to advancing this line of research. Using adult rodent models, Shirey and colleagues showed RSV A2 stimulation of primary cultured BALB/c BAL macrophage induced an early increase in CAM indicators, including iNOS, IL-12p40, and COX-2 [16]. Markers of AAM, Arginase-1, FIZZ1, and MR were constitutively elevated and increased later during viral infection, suggesting adult AM are responsible for both viral clearance and containment of a robust inflammatory response. In our neonatal mouse model, CAM was significantly impaired following RSV infection relative to adults. Rather, we observed an age-dependent increase in MR expression and uniform decrease in CCR7 expression through 10 dpi regardless of RSV infection (Fig. 5G–H). Increased MR expression with increasing age suggests the neonatal AM are achieving a resting steady state despite the added insult of RSV infection, likely due to an increased IL-4 to IFNγ ratio [37]. In the developing post-natal airway, this may serve as an important protective mechanism to avoid damaging inflammatory responses in lieu of added infection risks. In an attempt to balance the IL-4/IFNγ ratio in the RSV-infected neonatal airway, treatment of neonatal mice with i.n. rIFNγ significantly increased the CAM phenotype in RSV-infected neonatal mice without causing weight loss (Fig. 9); in fact, treatment moderately improved weight gain (Fig. 7). The increased CAM phenotype was greater in RSV-infected animals treated with IFNγ compared to mock-treated animals suggesting the effect was not due to IFNγ alone. Macrophage-derived cytokines, IL-12p40, IL-6, and TNFα were also increased in the RSV/rIFNγ+ compared control group indicating the combination of secondary stimulation with RSV induced greater activation than either virus or IFNγ alone (Fig. S2). Total neutrophil counts were significantly increased at 7 and 10 dpi in the RSV/rIFNγ+ group. Neutrophil infiltration has been implicated as a contributing factor in airway obstruction in severe RSV disease [4], thus some caution is warranted for consideration of IFNγ as a vaccine or therapeutic tool in infant RSV disease. Total lymphocytes were also increased by 10 dpi, however, this was well after RSV clearance had occurred suggesting lymphocytes played a minor, if any role in IFNγ-induced viral clearance in the neonatal mouse model (Fig. 8A). The reduction in viral load and improved weight gain increased in concert with CAM suggesting that i.n rIFNγ-induced macrophage activation contributed, at least in part, to viral clearance (Fig. 7 and 9).

We have demonstrated that neonatal AM are abundant in the neonatal BALB/cJ airway following RSV infection, similar to human infants, and their immature phenotype likely contributes to viral spread in the IFNγ-deficient lung environment. However, discriminating viral infection of AM versus phagocytosis of RSV or RSV-infected cells remains difficult. The pathology of acute RSV infection reported from four human infants showed that most AM were immunostain-positive for RSV suggesting AM associate with RSV *in vivo* during infection [31]. To better understand this association, we conducted functional and phenotypic studies *in vitro*. Our group, using J774A.1 macrophages (data not shown), and others using primary human cells, have shown that macrophages and monocytes are permissible to RSV and that permissibility is greater in cord blood monocytes>adult monocytes>adult macrophages, suggesting permissibility varies in relation to macrophage maturity [38]–[42]. In a culture environment, with no environmental signals, we showed J774A.1 macrophages differentiated to an AAM phenotype following RSV infection and mediated the spread of virus to adjacent Hep-2 cells. However, when J774A.1 macrophages were primed with IFNγ prior to infection, they differentiated to a CAM phenotype and the spread to Hep-2 cells was significantly reduced compared to cells that were not primed. It is conceivable that these *in vitro* studies closely model neonatal and adult airways following RSV infection in that the neonatal airway is devoid of IFNγ leaving the fate of the AM phenotype at the hands of RSV, which we showed differentiates to an AAM phenotype. Conversely, adult airways are rich in IFNγ during RSV infection, which lead to CAM differentiation capable of intracellular killing, pro-inflammatory cytokine secretion, and initiation of the adaptive immune response. These studies do not conclusively rule out the possibility that reductions in viral load *in vivo* may be partially attributable to the intrinsic anti-viral “interfering” properties of IFNγ. However, in our *in vitro* experiment design, RSV does not come in direct contact with IFNγ. Rather, J774A.1 macrophages are primed with IFNγ, the IFNγ solution is removed, and then the cells are infected. Additional control wells containing only RSV and rIFNγ showed no reduction in viral plaques, indicating that rIFNγ was activating macrophages to reduce the spread of infection to adjacent HEP-2 cells. Together, these data show rIFNγ stimulates macrophages to prevent the spread of RSV infection.

In summary, these data show the neonatal response to RSV infection depends on an abundant but immature AM population that fails to express a CAM phenotype in the IFNγ-deficient infant lung environment. Despite their age-dependent phenotype, treatment with i.n. IFNγ induces a CAM phenotype characterized by a significant reduction in MR, increases in MHC II, CD86, and CCR7, coupled with significant increases in the macrophage-derived cytokines, IL-12p40, IL-6, and TNFα. Treated mice showed a log-fold reduction in viral lung titers, expedited RSV clearance, and a significant increase in weight gain compared to untreated mice. Our *in vitro* data further supports our hypothesis that IFNγ-primed macrophages different to a CAM phenotype and reduce the spread RSV infection. Taken together, these data demonstrate i.n. IFNγ, a product that is already commercially available, is a viable treatment option and should be considered for further studies in neonatal RSV disease.

## Supporting Information

Figure S1 **Neonatal BALB/cJ mice express an age-dependent tissue macrophage phenotype following RSV infection.** Adult and pup BALB/cJ mice were infected with HD/HV RSV line19 or cell lysate. Cells were isolated from pup and adult digested lung tissue on 0, 2, 4, 7, and 10 dpi. The percent of immune cell subtypes were analyzed by flow cytometry in adults (A–D) pups (E–F), including MHC II (A, E), CD86 (B, F), CCR7 (C, G), and MR (D, H) on the CD11b− CD11c+ high gate. Mean values ± SD are depicted, and statistical difference was defined as a *P* value.05 for differences between mock-infected animals at the same time point (*); data are representative of two separate experiments.
(TIF)

Figure S2 **Pro-inflammatory cytokines are increased in RSV-infected neonatal mice following i.n. rIFN treatment.** Pup (2–4 days old) BALB/cJ mice received a HD/HV inoculum of RSV line 19 followed by 16 ng/g of i.n. rIFNγ (RSV/rIFNγ+) or diluent only (RSV/rIFNγ−) on 1, 3, 5, and 7 dpi. Control groups were mock-infected with cell lysate followed by 16 ng/g of i.n. rIFNγ (mock/rIFNγ+) or diluent only (mock/rIFNγ −) on 1, 3, 5, and 7 dpi. Pups were lavaged with 1.5–3 ml of cold HBSS/EDTA at the indicated dpi. The first wash of each lavage was reserved and frozen for analysis by luminex multiplex assay. Concentrations of IL-12p40 (A), IL-6 (B), and TNFα (C) were measured 4 and 7 dpi; all points are above the assay's LOQ. Mean values ± SD are depicted, and statistical difference was defined as a *P* value.05 for differences between groups at the same time point (*); data are representative of two separate experiments.
(TIF)
