# Supplementary figures and images for: Stimulation of Immature Lung Macrophages with Intranasal Interferon Gamma in a Novel Neonatal Mouse Model of Respiratory Syncytial Virus Infection
Source: PLoS One. 2012 Jul 6;7(7):e40499. doi: 10.1371/journal.pone.0040499 (PMC3391240; doi:10.1371/journal.pone.0040499)

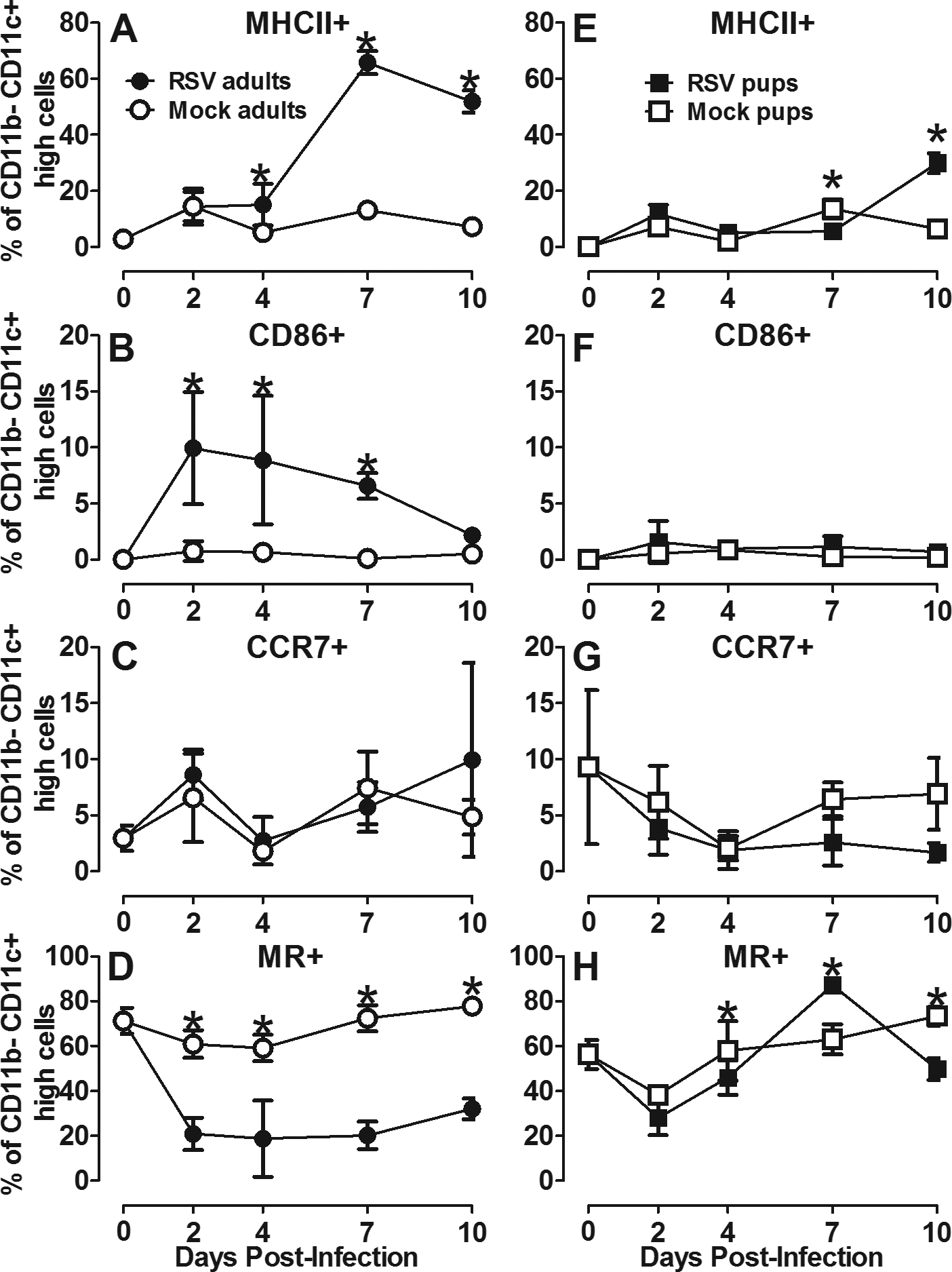

Supplement: Figure S1 — Neonatal BALB/cJ mice express an age-dependent tissue macrophage phenotype following RSV infection. Adult and pup BALB/cJ mice were infected with HD/HV RSV line19 or cell lysate. Cells were isolated from pup and adult digested lung tissue on 0, 2, 4, 7, and 10 dpi. The percent of immune cell subtypes were analyzed by flow cytometry in adults (A–D) pups (E–F), including MHC II (A, E), CD86 (B, F), CCR7 (C, G), and MR (D, H) on the CD11b− CD11c+ high gate. Mean values ± SD are depicted, and statistical difference was defined as a P value.05 for differences between mock-infected animals at the same time point (*); data are representative of two separate experiments. (TIF) [file pone.0040499.s001.tif]

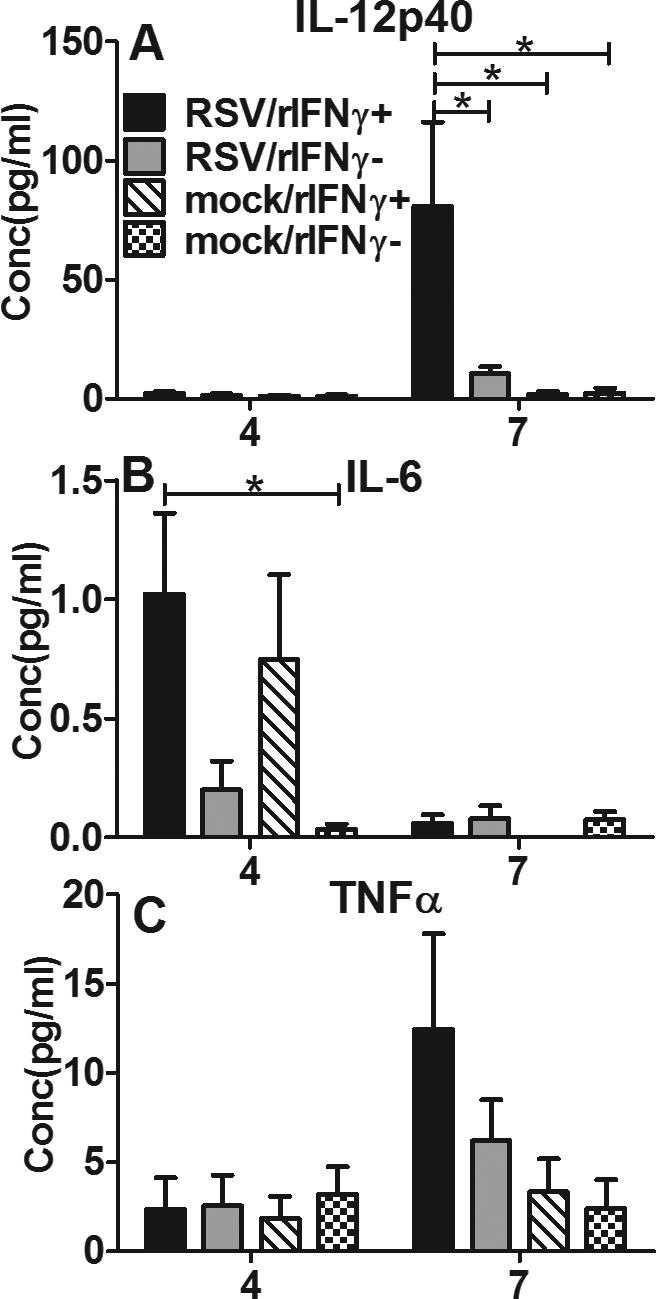

Supplement: Figure S2 — Pro-inflammatory cytokines are increased in RSV-infected neonatal mice following i.n. rIFN treatment. Pup (2–4 days old) BALB/cJ mice received a HD/HV inoculum of RSV line 19 followed by 16 ng/g of i.n. rIFNγ (RSV/rIFNγ+) or diluent only (RSV/rIFNγ−) on 1, 3, 5, and 7 dpi. Control groups were mock-infected with cell lysate followed by 16 ng/g of i.n. rIFNγ (mock/rIFNγ+) or diluent only (mock/rIFNγ −) on 1, 3, 5, and 7 dpi. Pups were lavaged with 1.5–3 ml of cold HBSS/EDTA at the indicated dpi. The first wash of each lavage was reserved and frozen for analysis by luminex multiplex assay. Concentrations of IL-12p40 (A), IL-6 (B), and TNFα (C) were measured 4 and 7 dpi; all points are above the assay's LOQ. Mean values ± SD are depicted, and statistical difference was defined as a P value.05 for differences between groups at the same time point (*); data are representative of two separate experiments. (TIF) [file pone.0040499.s002.tif]
